# Measures to Mitigate Sodium Valproate Use in Pregnant Women With Epilepsy

**DOI:** 10.7759/cureus.30144

**Published:** 2022-10-10

**Authors:** Saanthwana Ranjith, Abhishek Joshi

**Affiliations:** 1 Department of Obstetrics and Gynecology, Jawaharlal Nehru Medical College, Datta Meghe Institute of Medical Sciences, Wardha, IND; 2 Department of Community Medicine, Jawaharlal Nehru Medical College, Datta Meghe Institute of Medical Sciences, Wardha, IND

**Keywords:** awareness, mania, epilepsy, teratogenicity, complications, pregnancy, sodium valproate

## Abstract

Sodium valproate is a sodium salt of valproic acid. It is often used in the medical treatment of several conditions like epilepsy, bipolar disorder, mania, and migraines. This review debates whether the usage of valproic acid is appropriate in pregnancy. It also lists the various neonatal deformities and other teratogenic effects the said drug presents due to prenatal exposure to the drug and the implications of continuing drug therapy in certain situations. We should often weigh the outcomes and implement it only in conditions where its use is inevitable. It also includes the importance of awareness among middle-aged women with mental illness regarding the teratogenic effects of sodium valproate use and the relevance of discussion by physicians with patients regarding the usage of this drug despite being aware of the complications. It also explores other treatment options and modalities that can be used in the place of valproic acid for epilepsy and bipolar disorder in pregnant women and women of the reproductive age group, and how we can mitigate the usage of this drug by implementing various measures by referring to various guidelines present in different areas of the world. In summary, this article explores the numerous teratogenic effects sodium valproate presents in pregnancy, alternative medications, and treatment options instead of valproate. It also enumerates conditions where valproate use is necessary and how we can reduce and prevent the usage of valproate in pregnancy by opting for pregnancy prevention programs during valproate use and various other guidelines.

## Introduction and background

Sodium valproate is derived from the sodium salt of valproic acid. It is one of the most used antiepileptic drugs that are implemented today. Apart from its extensive use in epilepsy disorders, there are numerous clinical conditions where it is used. It is recently approved for various conditions that encompass medical therapy for indications like bipolar disorder [1]. It is also indicated for neuropathic pain, where small doses of the drug along with non-steroidal anti-inflammatory drugs have shown substantial results in the treatment of cervical and lumbar pain [2]. Extended-release (ER) divalproex sodium, which is a combination of valproic acid and its sodium salt derivative, has been indicated for the prophylaxis of migraine [3,4]. Seizure is a common phenomenon and is a condition of concern across all fields of medicine [5]. Epilepsy is a condition characterized by recurring seizures that are not provoked [6]. It is a neurological ailment found frequently in the population [7]. The occurrence of just one seizure does not substantiate the usage of antiepileptic drugs [8]. However, standard treatment involves prescribing various antiepileptic drugs according to the type and condition where epilepsy presents. For example, in generalized seizures, the medication of choice is valproic acid; however, it is administered at lower doses or not preferred in pregnancy [9]. Recent advances in the 21st century also suggest treatment options with neuromodulation techniques. Ablation using lasers has alleviated the frequency of seizures and epilepsy in a large population of those suffering from the condition [10].

A topic of relevance to this article is seizures observed in pregnancy. A point to note is that the frequency of seizures before and after pregnancy is less compared to that observed during pregnancy [11]. Physicians must also note the dangers present in a pregnant mother with epilepsy. Data from studies imply a 10 times greater mortality risk than mothers who did not have epilepsy [12]. The risks that the usage of anticonvulsant drugs poses compared to the risks of uncontrolled seizures are less dangerous [13]. Therefore, antiepileptic drugs are recommended despite being teratogens due to the hazardous nature of generalized tonic-clonic seizures to both the mother and fetus [14]. Some factors linked with seizures occurring in pregnancy are poor management and control of seizures previously, multidrug therapy with two or more drugs, and absence of treatment during pregnancy [15]. We must note that women with epilepsy should be evaluated and managed in an organized manner comprising many steps and may require combined effort and coordination with other specialties, including neurology and neonatology [16].

Hence, due to its high-risk profile, valproate is indicated for epilepsy only for females in the reproductive age group who are either not tolerant to it or to those who do not respond well to other medications that are prescribed for epilepsy (those who are more likely to suffer from puerperal psychosis or are more likely to relapse) [17] and to those who thoroughly follow a proper pregnancy prevention program. However, it is indicated as a first-line drug in women who have tested positively for HIV and are undergoing antiretroviral therapy along with lamotrigine [18]; the chief reason being that the rest of the drugs used for antiepileptic therapy may reduce the levels of antiretrovirals in the body by inducing p50 enzyme [19]. Therefore, women with epilepsy who are not resistant to other antiepileptic drugs are recommended alternative drug monotherapy with less dosage after considering its potential risks [20].

## Review

Sodium valproate in pregnancy

**Figure 1:**
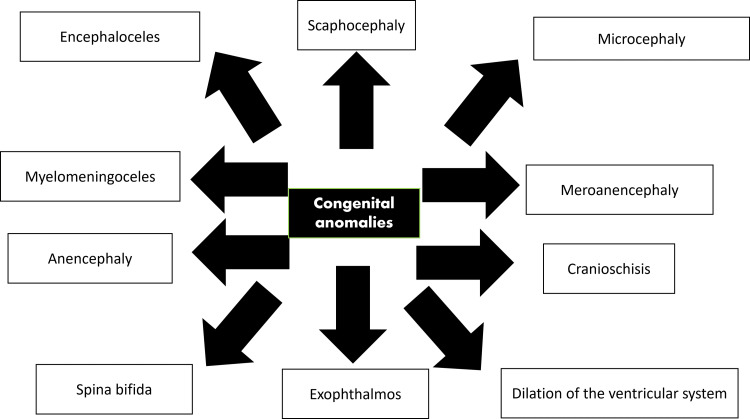
Most frequent anomalies observed congenitally in fetuses documented in a histochemical study of mice exposed to valproic acid Adapted from: Dupont S, Vercueil L: Epilepsy and pregnancy: What should the neurologists do? Rev Neurol (Paris). 2021, 177:168-79. 10.1016/j.neurol.2021.01.003 [30].

Valproic acid is linked to a spectrum of deformities, which includes a 20 times risk of acquiring neural tube deformities such as lumbosacral meningomyelocele (spina bifida aperta) [21], improper development of the lip, and palate (cleft) [22]. Abnormalities of the heart (atrial septal defects) are also present and are thought to be caused due to valproic acid inhibiting the enzyme histone deacetylase [23]. Telecanthus is also observed (soft tissues of intercanthi are widened), the upper lip is lengthened, and the philtrum is widened [24]. It is a matter of concern because of its apparent high risk in the causation of a low intelligence quotient and other neurodevelopmental conditions like autism and attention deficit hyperactivity disorder (ADHD) [25]. According to a cohort study conducted in Denmark, there is an association between exposure of neonates to valproate and the chance of the neonate to present disabilities intellectually and a chance of both childhood milestones arising late and disabilities that present intellectually [26]. Another study demonstrated that valproate use by mothers had produced offspring who demonstrated a significant reduction in performance at school while comparing children who were not exposed to antiepileptic medication and those whose mothers had reported lamotrigine use [27]. Prenatal antiepileptic medication exposure causes retardation of growth in infants in comparison to neonates who are not exposed to medication [28]. Some of the probable mechanisms responsible for the causation of fetal valproate syndrome disorders include programmed cell death and degeneration of neurons seen in the brain of rats (prenatally exposed to valproic acid) during development and increased plasticity of synapses shown in the medial prefrontal cortex of these rats, and reduction in folic acid levels. Implying that antioxidant defensive mechanisms are not enough, the oxidative stress that follows might be liable for damage observed in the brain apart from valproic acid [29]. Recent studies show that genetic factors might have a significant link to teratogenicity. If abnormalities were seen in the family previously, it enhances the risk. There are high chances of the second child acquiring abnormalities (about 17-36%) in conditions where the first child had abnormalities due to antiepileptic drug use [12]. Figure 1 summarizes some common anomalies that were observed in mice fetuses based on a histochemical study on prenatal valproic acid exposure [30].

Prevention and reduction of sodium valproate usage in pregnant patients who suffer from epilepsy and measures taken to mitigate the use of valproic acid in pregnancy

One thing that neurologists should be very cautious with and engaged in while caring for women who have epilepsy is considering the four concerns listed as follows: they should predict if there is a chance of the subject being pregnant, handle the risks of complications that are often present with the usage of antiepileptic drugs, and predict the risk of seizures and drug use during delivery and breastfeeding [31]. There are two hypothetical conditions where a female taking valproate will be in a quandary of resuming valproate use in pregnancy: the first one being if she is planning to be pregnant while undergoing sodium valproate therapy, and the second being if she becomes pregnant during therapy [32]. We can prevent the first condition by implementing a pregnancy prevention program comprising an evaluation that should assess her chances of conceiving before and during therapy. The female subject should be given information about the dangers valproic acid poses to their child who is yet to be born and the significance of proper contraceptive devices while on therapy. They should be analyzed by a specialist compulsorily every year, and they should also fill out a form that helps them to acknowledge the various risks present along with their uses. Drug packaging will have a pictorial presentation of the various dangers valproate presents in pregnancy. Pharmacists must describe the dangers it poses and provide a card that warns them regarding the same whenever they give the said medication to females of the reproductive age group [32].

Further guidelines to physicians regarding valproate use in pregnancy include those issued by the European Pharmacovigilance Risk Assessment Committee (PRAC) of the European Medicines Agency. They have advised stopping the usage of sodium valproate for treatment in pregnant women and of the childbearing age group due to the risk of acquiring various defects and neurodevelopmental conditions in offspring. It included the following suggestions: valproic acid should not be considered for treating epilepsy or bipolar disorder in either of the previously mentioned groups. They should only be considered when other medications are ineffective or cannot be tolerated by the subject. Effective contraception techniques must be implemented, and the beginning and the course of the medication must be handled by medical practitioners accustomed to treating such diseases. The doctors advising its use to the reproductive age group must provide comprehensive data about the various risks it presents to ensure that the decision is made after understanding said information and its complications. Females in valproic acid therapy should not cease their medication intake without referring their medical practitioner. It should be avoided in the treatment of migraines [25].

The second scenario may also present due to the failure of certain oral contraceptive drugs while on antiepileptic therapy with certain antiepileptic drugs, which are grouped into different categories: the first one being classical enzyme inducers, i.e., carbamazepine, phenytoin, and phenobarbitone increase the breakdown of estrogen and progesterone that are administered orally. Hence, various formulations like progestin-only pills, contraceptives that consist of estrogen and progestin combinations, and subdermal progestin pills prove ineffective. The second category includes antiepileptic drugs whose enzyme-inducing capacity depends upon the dosage; this includes topiramate and perampanel; enzyme induction is less potent than the first category. However, they cause a reduction in plasma concentration levels of local and oral contraceptives. Hence, these are not indicated for use along with these drugs. The third category includes antiepileptic drugs, which induce enzymes like felbamate and rufinamide (at higher doses), both decrease levels of hormonal contraceptives by increasing metabolism. However, enzyme inhibitors like sodium valproate do not interfere with the metabolism of oral formulations of estrogen and progesterone. Neutral antiepileptic drugs like levetiracetam and lamotrigine do not supposedly interfere with the breakdown of oral contraceptives [31].

We have to explore alternative treatments for epilepsy in pregnancy in place of sodium valproate to reduce its usage and its congenital morbidities. Other treatment strategies that can be implemented instead of sodium valproate can be evaluated by assessing the teratogenic risks of other antiepileptic drugs and opting for treatment options that pose fewer complications for the unborn baby. In the single-drug treatment of epilepsy, the highest incidence of significant congenital deformities is seen with sodium valproate, relatively moderate rates are seen with topiramate and phenobarbital, and an intermediate incidence is observed in carbamazepine, oxcarbazepine, and phenytoin. The lowest incidence is observed with levetiracetam and lamotrigine. According to the North American antiepileptic pregnancy registry, all the previously mentioned drugs have a higher risk than the internal control rate of 1.2%. An increased dosage of sodium valproate and topiramate is associated with even more incidence of significant congenital morbidities. Multidrug therapy, including medications like sodium valproate and topiramate, results in a higher incidence of significant morbidities [33]. Assessing risk patterns of other antiepileptics by referring to consistent research studies, the least number of malformations are observed with lamotrigine and levetiracetam, which include no or slight incidences of structural deformities, no organ-centric deformities except for an increased incidence of oral clefts in single drug therapy of lamotrigine, but this is not observed in all studies. Concerning the dose that must be administered, it is suggested to stick to the smallest dosage appropriate for properly managing seizures. However, proper monitoring should be done with said drugs because of the high rates of clearance of both drugs [34]. Hence, for treating epilepsy, these two are favored compared to the risks that the other antiepileptic drugs present.

Some other strategies of mitigating sodium valproate use by other medications are demonstrated in Western Cape province, where they performed various measures to prevent valproate use by increasing the availability of alternative drugs that are comparatively safer options in pregnancy; these include lamotrigine, clobazam (for a short interval of time to cover the slow dose - escalation when lamotrigine therapy is started), and levetiracetam. However, in the case of levetiracetam, the restriction guidelines for the physician and the subject undergoing therapy are reduced. Other strategies include instructing registered physicians who prescribe these drugs by district specialists with extensive clinical knowledge on the side effects of various antiepileptic drugs present and a regimen that involves titration to aid females undergoing valproate therapy to transition to lamotrigine, issuing a provincial document that gives clinical guidelines to heads of units of neurology and psychiatry on the most efficient usage of drugs in women who have epilepsy and bipolar mood disorder. They also provide a risk acknowledgment form for completion by medical practitioners who prescribe medications and those women who have started their treatment with valproic acid every year after the first year of initiation of treatment [18]. The guidelines from the Centre of Perinatal Excellence and the National Institute for Health and Care Excellence UK include not providing valproate prescriptions to females who belong to the age group who can bear children or considering medication if a pregnancy prevention strategy is implemented. Women planning to conceive while on valproic acid therapy lessen valproate use for two to four weeks, in addition to a high intake of folic acid to follow up in the first trimester. In case of pregnancy, stop treatment that involves using valproate, consider risk factors, and prescribe other medical alternatives for anti-convulsive therapy with great care and seek advice from psychiatric consultants. Measures also include monitoring the infant closely and consulting a medical specialist in neonatology wherever possible. The primary reason for valproate use during pregnancy by patients is a lack of awareness about teratogenicity among pregnant women or women of reproductive age group, and this is demonstrated in Table 1 below, which is based on a study conducted in a group of 23 individuals [35].

**Table 1:** Awareness of congenital abnormalities that occur with valproate use Adapted from: Sibanyoni AU, Joubert M, Naidu K: Are female bipolar patients of reproductive age aware of the teratogenic risk of sodium valproate? A qualitative study. South Afr J Psychiatry. 2022, 28:1719. 10.4102/sajpsychiatry.v28i0.1719 [35].

Awareness of congenital abnormalities that occur with valproate use	Number of participants in the study	If aware, the channel of information
Not aware	4/23	-
Slightly understand valproate use might endanger the child, but have not received formal counseling	6/23	Informally acquired knowledge from friend circles or family
Has complete knowledge regarding teratogenicity sodium valproate presents	13/23	Have acquired information from the physician or their own research

However, irrespective of their awareness regarding sodium valproate, 20/23 participants knew that when they get pregnant while on valproate therapy, there are different steps to be followed accordingly, like changing medication and decreasing dosage. Therefore, this lack of awareness mandates discussion by the physicians with the patient regarding the use of valproic acid. Table 2 discusses the common questions posed by the patient regarding valproate use in pregnancy and how the doctor can answer these questions [31].

**Table 2:** Importance of discussion between physicians and patients and questions posed regarding valproate use Adapted from: Macfarlane A, Greenhalgh T: Sodium valproate in pregnancy: what are the risks and should we use a shared decision-making approach? BMC Pregnancy Childbirth. 2018, 18:200. 10.1186/s12884-018-1842-x [31].

Questions that the patient often poses	Continuation of valproate use with the same amount of dosage	Decreasing the dosage of valproic acid	Stopping the valproic acid medication	Switching to another antiepileptic medication
The patient asks questions regarding the involvement	Same as before	Is done over a time frame of weeks/months	It is done after a certain time frame of lowering the dosage	Usually involves switching to antipsychotics or safer antiepileptics like lamotrigine
Asks about the dangers valproic acid poses to the subject	Side effects include loss of hair, PCOS in females, and other side effects of valproate use	Side effects are usually seen, and more chances of relapse	Greater chances for relapse to occur and increased rates of occurrence of puerperal psychosis	New adverse effects presented by replacement drugs, increased chances of relapse if the drug is not as efficient as valproic acid
Advantages in presuming valproate use	Fewer chances of the subject relapsing and fewer chances of puerperal psychosis	Fewer chances of deformities due to less prenatal exposure	The same percentage of chances of observing deformities in the child as that seen in the general population	If the new drug is tolerable, fewer chances of attacks reoccurring
Asks about dangers posed to the child	Congenital deformities and developmental	Lesser chances of deformities	Can indirectly affect the child, improper inhibition of bipolar disorder, and relapse of epilepsy attacks in the mother can cause hypoxia	Some medications pose fewer risks to the child
The population that would receive the maximum number of advantages in each situation	Persons whose bipolar disorder is not stable, people where recurrence of episodes is more	Persons whose bipolar disorder cannot be treated with other medication	Persons who are stable without valproate use and do not desire to take other drugs during the period of pregnancy	People where another medication is effective as a replacement for valproate

Therefore, in retrospect, we realize the importance of proper communication between physicians and patients regarding valproate use. It is mandatory to increase awareness and prevent the unnecessary use of valproate during pregnancy by letting the patient fill out an informed consent form by weighing the pros and cons of its use.

A study in five European countries compared valproate prescriptions before and after implementing risk minimization measures in outpatient settings in 2014. According to this study, valproate initiation as second-line therapy differed across various countries, implying less effectiveness on specific grounds. However, the positive effects of these measures include a reduced number of valproate prescriptions in pregnant women and a reduced incidence of pregnancies exposed to valproate. On these grounds, the European Medical Association introduced extra measures to enforce the previous restrictions. Further studies are underway to determine the effectiveness of the new measures and to monitor valproate use in women with childbearing potential and pregnancies exposed to it [36].

In summary, for the management of a woman with epilepsy before, during, and after pregnancy, including when a woman with epilepsy is planning for pregnancy, we should choose the medication with the lowest risk of teratogenicity. We need to establish a baseline dose for each individual and titrate it to the lowest effective dose. We should prefer monotherapy over polytherapy. Medications like lamotrigine and levetiracetam are preferred compared to drugs like valproate. A high amount of folic acid is recommended in pregnancy, especially when using sodium valproate and enzyme-inducer antiepileptic drugs. During pregnancy, for stable seizures, a minimum of three visits are suggested. If not, then more frequent visits are required. We should monitor antiepileptic drug serum levels and adjust the dosage if there is a decrease in the serum levels or an increased frequency of seizures. Prenatal ultrasonographic organ screening is recommended during the 19th to 21st gestational week. Vaginal delivery is generally preferred. A cesarean section is usually recommended in cases with a high risk for seizures or poor seizure control during labor. In the puerperium period, drug monitoring is recommended in the first week to adjust the dosage. Sleep deprivation is expected in the postpartum period, and sleep deprivation is often correlated with increased seizures. A higher dose than the preconception period may be advised for this reason. Breastfeeding is highly recommended [37].

## Conclusions

This article summarizes the broad spectrum of uses of valproate and the various implications that valproate use and disuse present in the fetus and the mother. It also demonstrates why physicians should implore effective use of antiepileptic drugs and drugs for bipolar disorders in women of reproductive age group or those with reproductive potential by weighing outcomes in both mother and child and should refrain from valproate use unless or until necessary. Antiepileptic drugs that pose less harm in pregnancy, like lamotrigine or levetiracetam, are preferred instead. It is also essential to consider the mother's decision to resume treatment with valproate or other medication or reduce the dosage. However, it is the duty of the physicians who prescribe such medication to inform the mother about the risks it poses to the child. Physicians can reduce valproate use by implementing various programs such as the pregnancy prevention program in the UK (care should be taken not to use antiepileptic drugs that are enzyme inducers that promote oral contraceptive failure) and measures directing lamotrigine use as per guidelines followed in districts of Cape Town, South Africa (along with other measures), and various guidelines issued by the PRAC. It is imperative to determine and monitor the effectiveness of these guidelines and measures in pregnant women and women with childbearing potential. The article also adds a small note regarding the management of antiepileptic drug use before, during, and after pregnancy.
